# Role of Zerumbone, a Phytochemical Sesquiterpenoid from *Zingiber zerumbet Smith*, in Maintaining Macrophage Polarization and Redox Homeostasis

**DOI:** 10.3390/nu14245402

**Published:** 2022-12-19

**Authors:** Wei-Lan Yeh, Bor-Ren Huang, Guan-Wei Chen, Vichuda Charoensaensuk, Cheng-Fang Tsai, Liang-Yo Yang, Dah-Yuu Lu, Mao-Kai Chen, Chingju Lin

**Affiliations:** 1 Department of Biochemistry, School of Medicine, China Medical University, Taichung 40402, Taiwan; 2 Institute of Translational Medicine and New Drug Development, China Medical University, Taichung 40402, Taiwan; 3 Department of Neurosurgery, Taichung Tzu Chi Hospital, Buddhist Tzu Chi Medical Foundation, Taichung 427213, Taiwan; 4 School of Medicine, Tzu Chi University, Hualien 97004, Taiwan; 5 Department of Pharmacology, School of Medicine, College of Medicine, China Medical University, Taichung 40402, Taiwan; 6 Department of Physiology, School of Medicine, China Medical University, Taichung 40402, Taiwan; 7 Department of Medical Laboratory Science and Biotechnology, Asia University, Taichung 41354, Taiwan; 8 Laboratory for Neural Repair, China Medical University Hospital, Taichung 404327, Taiwan; 9 Department of Photonics and Communication Engineering, Asia University, Taichung 41354, Taiwan; 10 Department of Anesthesiology, China Medical University Hospital, Taichung 404327, Taiwan

**Keywords:** zerumbone, microglial cells, macrophage polarization, neuroinflammation, redox homeostasis

## Abstract

Macrophages and microglia are highly versatile cells that can be polarized into M1 and M2 phenotypes in response to diverse environmental stimuli, thus exhibiting different biological functions. In the central nervous system, activated resident macrophages and microglial cells trigger the production of proinflammatory mediators that contribute to neurodegenerative diseases and psychiatric disorders. Therefore, modulating the activation of macrophages and microglia by optimizing the inflammatory environment is beneficial for disease management. Several naturally occurring compounds have been reported to have anti-inflammatory and neuroprotective properties. Zerumbone is a phytochemical sesquiterpenoid and also a cyclic ketone isolated from Zingiber zerumbet Smith. In this study, we found that zerumbone effectively reduced the expression of lipocalin-2 in macrophages and microglial cell lines. Lipocalin-2, also known as neutrophil gelatinase-associated lipocalin (NGAL), has been characterized as an adipokine/cytokine implicated in inflammation. Moreover, supplement with zerumbone inhibited reactive oxygen species production. Phagocytic activity was decreased following the zerumbone supplement. In addition, the zerumbone supplement remarkably reduced the production of M1-polarization-associated chemokines CXC10 and CCL-2, as well as M1-polarization-associated cytokines interleukin (IL)-6, IL-1β, and tumor necrosis factor-α. Furthermore, the expression of inducible nitric oxide synthase (iNOS) and cyclooxygenase-2 and the production of NO were attenuated in macrophages and microglial cells supplemented with zerumbone. Notably, we discovered that zerumbone effectively promoted the production of the endogenous antioxidants heme oxygenase-1, glutamate–cysteine ligase modifier subunit, glutamate–cysteine ligase catalytic subunit, and NAD(P)H quinone oxidoreductase-1 and remarkably enhanced IL-10, a marker of M2 macrophage polarization. Endogenous antioxidant production and M2 macrophage polarization were increased through activation of the AMPK/Akt and Akt/GSK3 signaling pathways. In summary, this study demonstrated the protective role of zerumbone in maintaining M1 and M2 polarization homeostasis by decreasing inflammatory responses and enhancing the production of endogenous antioxidants in both macrophages and microglia cells. This study suggests that zerumbone can be used as a potential therapeutic drug for the supplement of neuroinflammatory diseases.

## 1. Introduction

Macrophages are key immune cells that maintain homeostasis and defense during periods of both good health and disease by regulating the onset and resolution of inflammation [1]. Tissue macrophages reside in almost every part of the human body, including the brain. Resident microglia in the central nervous system (CNS) are local phagocytic cells that mediate immunological and inflammatory reactions in response to pattern-associated molecular patterns (PAMPs) and damage-associated molecular patterns (DAMPs) in the environment [2]. Several environmental factors alter the phenotype of macrophages, thereby affecting their functions. Macrophages can be polarized into M1-like and M2-like phenotypes [3]. Typically, microglia in healthy brain tissues are in a quiescent M2 phenotype, and these microglia are critical for the maintenance of neuron–microglia interactions and neuronal development [4]. M1 macrophages are activated by bacterial lipopolysaccharide (LPS) and proinflammatory cytokines, including tumor necrosis factor (TNF) and interferon (IFN). Activated M1 macrophages overexpress inducible nitric oxide synthase (iNOS), nitric oxide (NO), and reactive oxygen species (ROS) and upregulate proinflammatory mediators such as interleukin (IL)-1β, IL-6, and TNF-α [5,6,7]. ROS, which are produced in response to oxidative and environmental stress, cause the activation of microglia [2]. Activated M1 microglia produce various proinflammatory mediators and free radicals that inhibit brain repair and regeneration, leading to neuroinflammation, neurodegenerative diseases, and psychiatric disorders [8]. Moreover, M1 macrophages produce several chemokines, such as the C–X–C motif chemokine ligand (CXCL)-10 [9] and C–C motif chemokine ligand (CCL)-2 [10], which trigger the activation of type 1 T helper (Th1) response, accelerate phagocytic activity, and promote inflammation [6]. By contrast, M2 macrophages are activated in response to the Th2 response. Upregulation of arginase-1 (Arg-1) and IL-10 in M2 macrophages promotes cell proliferation, tissue repair, and anti-inflammatory cytokines, thereby counteracting the inflammation triggered by activated M1 macrophages [11]. M2 microglia increase brain repair and regeneration by promoting phagocytosis, producing endogenous trophic factors, and alleviating brain inflammation [1]. However, disruption in the homeostasis of M1 versus M2 phenotypes results in the development of several diseases, including obesity, atherosclerosis, and insulin resistance [12].

Generally, the proportion of M1 and M2 macrophages is tightly controlled in healthy tissues [5]. Substantial evidence has been obtained indicating that the modulation of macrophage polarization plays a crucial role in the pathology of several diseases, including obesity [13], atherosclerosis [14], and cancers [15]. According to a study by Jiang et al., spinal cord injury (SCI) induced the expression of M1 phenotypic markers (CD86, iNOS, IL-6, and TNF-α) and decreased the expression of M2 phenotypic markers (CD206, IL-10, and Arg-1) [16]. In addition, treatment with substance P improved recovery from SCI by inducing the production of endogenous anti-inflammatory mediators [16]. Our previous study showed that treatment with paliperidone effectively decreased the expression of an M2 phenotype marker (CD206) while increasing that of an M1 phenotype marker (CD80), resulting in the inhibition of glioblastoma and suggesting that regulation of macrophage polarization is a potential treatment strategy for certain diseases [17]. Notably, our recent findings indicate that inhibiting lipocalin-2 expression in macrophages and microglial cells may be a novel strategy for the treatment of neuroinflammation and neurodegenerative diseases [18]. Lipocalin-2 has been characterized as an adipokine/cytokine and was found to be associated with several cellular processes, including cell survival, death, differentiation, invasion, migration, inflammatory response, iron homeostasis, insulin resistance, and tissue regeneration [19]. An increased level of lipocalin-2 expression was correlated with acute and chronic liver injury [20]. Following acute inflammation, the liver overexpresses lipocalin-2, triggering inflammatory cell infiltration for phagocytosis and ensuring homeostasis [21]. One study discovered that lipocalin-2 promoted microglial M1 polarization, resulting in impairment of cognitive function and motor behavior due to neuroinflammation [22]. A recent study reported that the neutralization of lipocalin-2 diminishes the severity of brain injury caused by ischemia reperfusion [23]. Moreover, lipocalin-2-deficient mice exhibited a weaker M1 phenotype with an increase in the strength of the M2 phenotype, suggesting that lipocalin-2 plays a critical role in microglial polarization [22].

Zerumbone is a dietary compound presented in a variety of natural foods. It is naturally occurring in plants of the *Zingiberaceae* and *Curcuma* families, notably *Zingiber zerumbet* Smith, and features as a monocyclic sesquiterpene phytochemical [24]. Zerumbone has been reported to possess diverse biological activities, including activities against microbes, osteoporosis, prostatic hyperplasia, and polycystic ovary syndrome [24,25]. The safety, cytotoxicity, and chemopreventive potential of zerumbone have been reported [26,27,28]. Zerumbone was also reported to possess anti-inflammatory effects against acute and chronic inflammation of granulomatous tissue in mice [29]. In addition, oral administration of zerumbone did not result in any clinical abnormalities or other adverse effects in one study [30]. Zerumbone has been discovered to possess anti-inflammatory and antioxidant activities in various inflammation-related diseases [31,32]. Additionally, zerumbone was found to be beneficial for the treatment of learning and memory impairment in an animal model [33]. A recent study suggested that zerumbone decreased proinflammatory cytokine expression, β-amyloid production, and behavioral deficits in APP/PS1 transgenic mice [34]. However, the effects (and underlying mechanisms) of zerumbone on lipocalin 2 expression and macrophage polarization, as well as the generation of endogenous antioxidant enzymes and anti-inflammatory proteins in the CNS, remain poorly understood.

This study aimed to elucidate the regulatory effects of zerumbone on homeostasis and M1/M2 macrophage polarization by considering lipocalin-2 expression, oxidation/antioxidation effects, and inflammatory/anti-inflammatory effects in macrophages and microglial cells. Furthermore, this study investigated the effect of zerumbone on the activity of endogenous antioxidants and anti-inflammatory proteins in macrophages and microglial cells. In summary, this study suggests that zerumbone may be a potential supplement for inflammatory diseases and neurodegenerative diseases.

## 2. Materials and Methods

### 2.1. Materials

Primary antibodies against GSK3α/β, β-actin, and phosphor-Akt^Ser743^ were obtained from Santa Cruz Biotechnology (Santa Cruz, CA, USA). Antibodies against phosphor-AMPK^Thr172^ and phosphor-GSK3α/β^Ser21/Ser9^ were purchased from Cell Signaling Technology. Anti-iNOS antibody (610431) was acquired from BD Transduction Laboratories (Lexington, KY, USA). Cyclooxygenase (COX)-2 polyclonal antibody (aa 570–598) was purchased from Cayman Chemicals (Ann Arbor, MI, USA). Heme oxygenase (HO)-1 polyclonal antibody was obtained from Enzo Life Sciences Inc. (Farmingdale, NY, USA). Antibodies against glyceraldehyde-3-phosphate dehydrogenase (GAPDH; GCLC, GCLM, and NQO1) were acquired from Abcam (Cambridge, MA, USA).

### 2.2. Cell Culture

In a humidified incubator containing 5% CO_2_ and 95% air at 37 °C, mouse macrophages RAW264.7 cells were cultured in high glucose Dulbecco’s modified Eagle’s medium (DMEM), 10% fetal bovine serum (FBS), and 100 U/mL penicillin/streptomycin. The adult mouse microglia (IMG) was obtained from the Harvard School of Public Health (Boston, MA, USA). IMG cells expressing a microglial-specific marker represent brain microglia features morphologically and functionally. The IMG cells were cultured in DMEM with low glucose content (1 g/L), 10% FBS, and 100 U/mL penicillin/streptomycin.

### 2.3. Western Blotting Analysis

The cells were lysed on ice for 30 min with radioimmunoprecipitation assay buffer containing a protease inhibitor cocktail. The supernatant was collected after centrifugation, and proteins in the supernatant were separated using sodium dodecyl sulfate–polyacrylamide gel electrophoresis. The blots were transferred onto polyvinylidene fluoride membranes. After being blocked with nonfat milk, the membranes were probed with primary antibodies and secondary antibodies. Proteins were visualized through enhanced chemiluminescence using Kodak X-OMAT LS film (Eastman Kodak, Rochester, NY, USA). The densitometric values were quantified by ImageJ software.

### 2.4. NO Assay

The NO assay method is described in our previous publication [35]. Briefly, culture supernatant containing nitrite was reacted for 10 min with 0.1% NED solution and 1% sulfanilamide in 5% phosphoric acid avoiding light. NO was quantified by measuring the amount of nitrite under OD 520 nm using a microplate reader.

### 2.5. Quantitative Real-Time Polymerase Chain Reaction (PCR)

mRNA levels were detected using quantitative real-time PCR, and total RNA was extracted using TRIzol reagent (Invitrogen, Carlsbad, CA, USA). An amount of 2 μg of total RNA was used for reverse transcription (RT) by using an RT Kit (Invitrogen, Carlsbad, CA, USA). SYBR Green Master Mixes (Applied Biosystems, Waltham, MA, USA) was used for conducting PCR. To calculate the transcripts cycle (denoted CT), the threshold was set within the linear phase of gene amplification.

### 2.6. Phagocytosis Assay

The phagocytosis assay method was performed in accordance with the method in our previous study [35]. The cells were seeded onto culture dishes and grown at 37 °C and 5% CO_2_. After drug treatment, the medium was replaced with medium containing carboxylate-modified polystyrene fluorescent yellow–green latex beads (YG beads; Cat#L4655; Sigma Aldrich, St. Louis, MA, USA), and the cells were incubated at 37 °C. The cells were trypsinized after several washes to remove the noninternalized beads, and their phagocytic activity was quantified using flow cytometry.

### 2.7. Statistical Analysis

GraphPad Prism 6.0 (Graph Pad Software, San Diego, CA, USA) was used for statistical analysis. Values are presented as the mean ± standard error of the mean (SEM). Significance of the differences between the groups was analyzed by Student’s *t*-test. One-way analysis of variance (ANOVA) with the Bonferroni post hoc test was used for comparisons of more than two groups. A *p* < 0.05 was considered significant.

## 3. Results

### 3.1. Zerumbone Lowers the Expression of Lipocalin-2 in Macrophages and Microglial Cells

**Figure 1:**
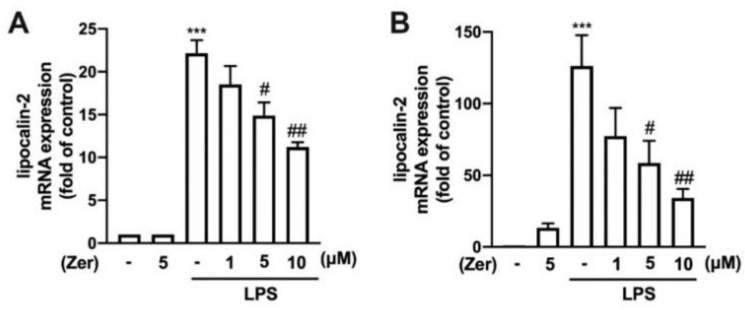
Inhibitory effects of zerumbone on the expression of lipocalin-2 in macrophages and microglia. RAW264.7 macrophages (**A**) and IMG adult mouse microglial cell lines (**B**) were supplemented with different concentrations of zerumbone (1, 5, or 10 µM) for 30 min and administered with lipopolysaccharide (LPS; 50 ng/mL) for 6 h. Lipocalin-2 mRNA expression levels were determined using real-time PCR and normalized to β-actin. Data are presented as the mean ± standard error of the mean (SEM) (*n* = 3 or 4). *** *p* < 0.005 compared with the control group. # *p* < 0.05, ## *p* < 0.01 compared with the LPS alone group.

RAW264.7 mouse macrophages (Figure S1) and IMG adult mouse microglia (Figure S2) were supplemented with zerumbone (1, 5, or 10 µM), and no toxicity was then observed. As shown in Figure 1, supplement with zerumbone alone did not affect the expression of lipocalin-2 in either cell model. Application of LPS resulted in significantly increased lipocalin-2 expression in both the macrophages (Figure 1A) and microglia (Figure 1B). Moreover, the zerumbone supplement effectively decreased LPS-stimulated lipocalin-2 expression in a concentration-dependent manner for macrophages (Figure 1A) and microglia (Figure 1B), with a 40% and 75% reduction under the maximum concentration of zerumbone.

### 3.2. Supplement with Zerumbone Decreases H_2_O_2_, ROO•, and HO• Production in Microglial Cells

**Figure 2:**
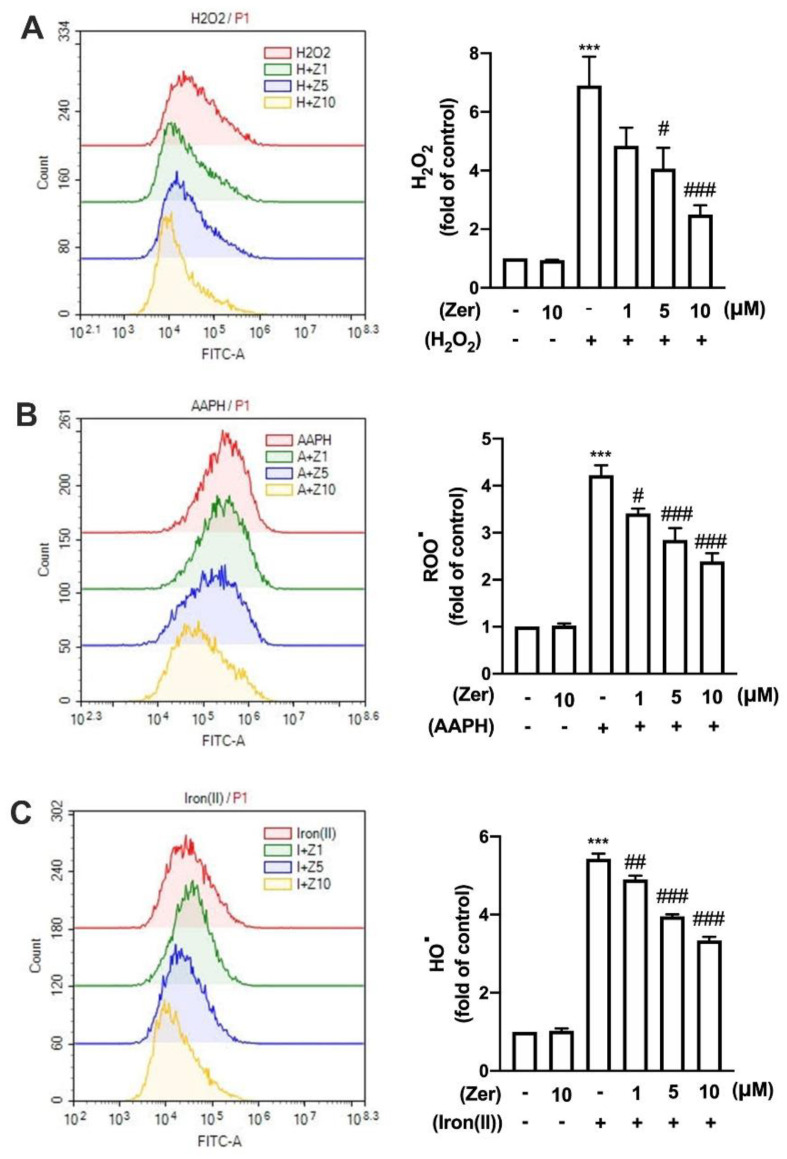
Effects of zerumbone on ROS production in microglia. IMG cells were supplemented with various concentrations of zerumbone (1, 5, or 10 μM) for 30 min, followed by 5 mM H_2_O_2_ (**A**), 5 mM AAPH (**B**), or 1 mM iron (ll) with 0.5 mM H_2_O_2_ (**C**) for another 90 min. The intensity of dichlorofluorescein (DCF) fluorescence was detected through flow cytometry after 40 min of incubation with 10 µM dichloro-dihydro-fluorescein diacetate (DCFH-DA). Quantitative data are represented as the mean ± SEM (*n* = 4). *** *p* < 0.005 compared with the control group. # *p* < 0.05, ## *p* < 0.01, ### *p* < 0.005 compared with the treatment group alone.

The microglial cells were treated with either hydrogen peroxide (H_2_O_2_), 2, 2′-azobis (2-amidinopropane) hydrochloride (AAPH), or iron (II) plus H_2_O_2_ to stimulate the production of various ROS. Then, the effects of zerumbone on ROS production were determined. As illustrated in Figure 2, supplement with zerumbone alone did not influence ROS production. H_2_O_2_, AAPH, and iron resulted in ROS levels in microglial cells that were approximately four- to six-fold higher than those in the control group. Notably, zerumbone decreased H_2_O_2_ production in a concentration-dependent manner (Figure 2A). Supplement with zerumbone further decreased AAPH-induced peroxyl radical (ROO•) production (Figure 2B). Moreover, hydroxyl radical (HO•) production stimulated by iron (II) and H_2_O_2_ following the zerumbone supplement was markedly decreased in a concentration-dependent manner (Figure 2C). This study suggests that supplements with zerumbone concentration-dependently inhibited H_2_O_2_, ROO•, and HO• production in microglial cells.

### 3.3. Inhibitory Effect of Zerumbone against Phagocytic Activity in Microglial Cells

**Figure 3:**
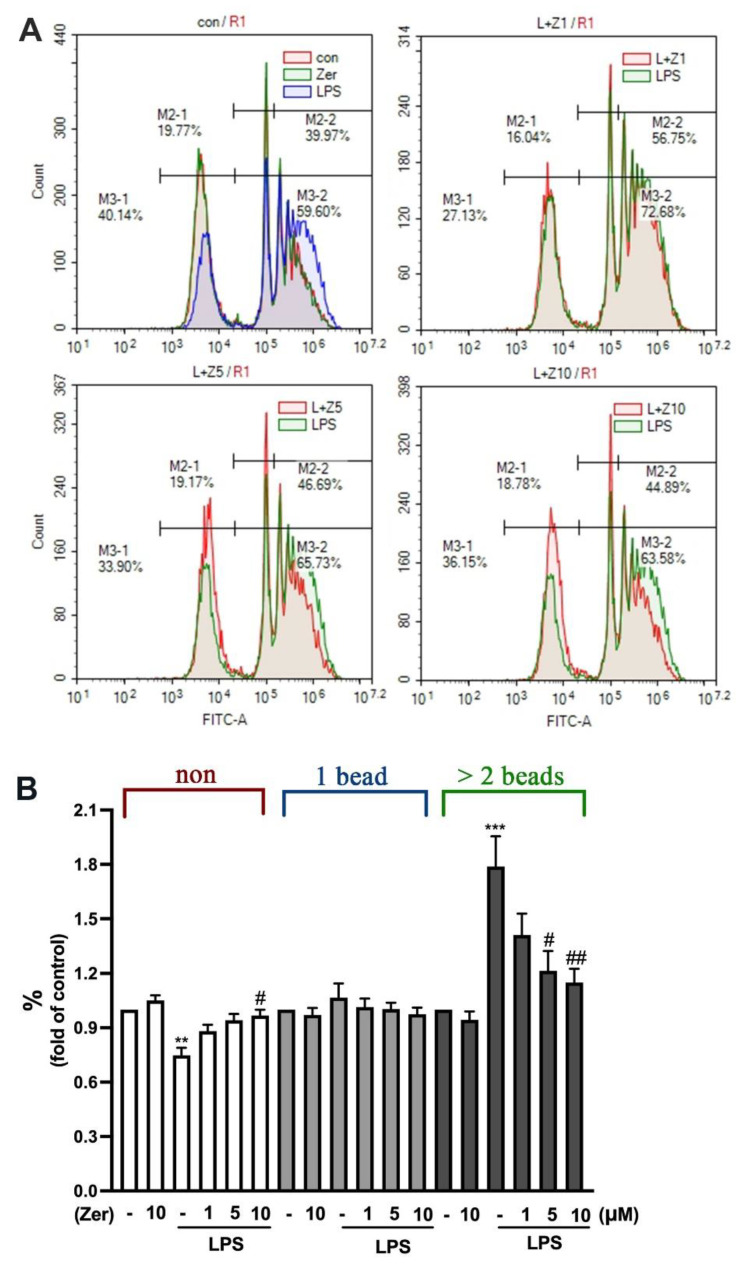
Effect of zerumbone on phagocytic ability in microglia. (**A**) IMG cells were presupplemented with different concentrations of zerumbone (1, 5, or 10 μM) for 30 min and LPS (50 ng/mL) for another 24 h. After incubation of the cells with 1 μm fluorescent YG beads for 1 h at 37 °C, the intensity of the beads was analyzed using flow cytometry. The quantitative results shown in (**B**) are the mean ± SEM (*n* = 4): non, no bead was uptaken by cell; 1 bead, cell uptake 1 bead; >2 beads, cell uptake more than 2 beads. ** *p* < 0.01, *** *p* < 0.005 compared with the control group. # *p* < 0.05, ## *p* < 0.01 compared with the LPS alone group.

We further investigated the effect of zerumbone on phagocytosis in microglial cells. The results revealed that the nonphagocytic populations were remarkably smaller in the LPS-activated microglial cells than in the non-LPS-activated microglial cells (Figure 3). However, the phagocytic populations that engulfed two or more beads were larger. Furthermore, supplement with zerumbone alone did not change the ability of phagocytosis of microglial cells either in one or in two or more beads (Figure 3A upper-left panel, B). Notably, 1 µM zerumbone slightly decreased LPS-stimulated phagocytosis in microglial cells (Figure 3A upper-right panel, B). In addition, zerumbone at higher concentrations (5 and 10 µM) effectively decreased the phagocytic populations engulfing two or more beads in the LPS-stimulated microglial cells (Figure 3A lower panel, B). These results confirm that supplement with zerumbone alone did not affect the ability of phagocytosis. Moreover, zerumbone effectively inhibited LPS-stimulated microglial phagocytosis.

### 3.4. Zerumbone Reduces the Expression of Proinflammatory Mediators and M1-Macrophage Polarization Markers in Macrophages and Microglial Cells

**Figure 4:**
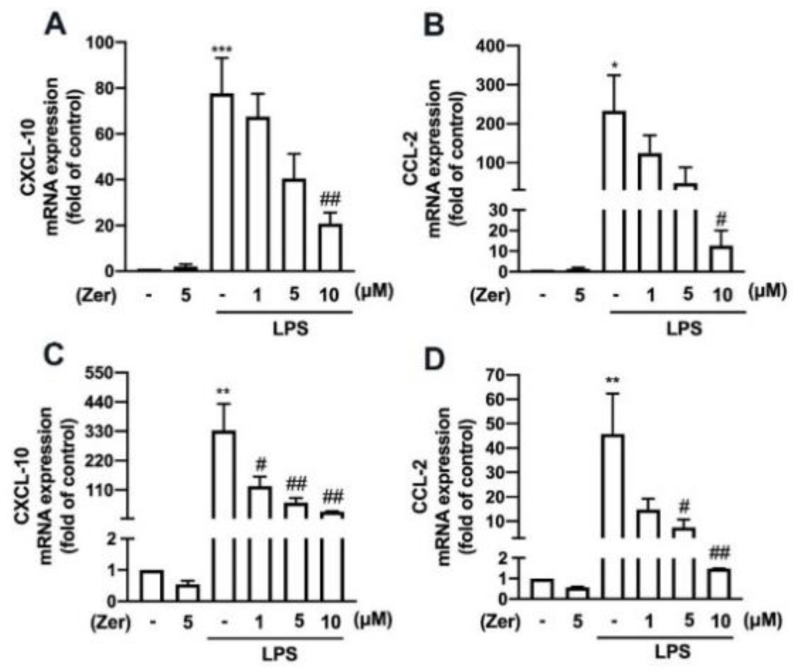
The expression of proinflammatory mediators in macrophages and microglia in response to zerumbone. RAW264.7 (**A**,**B**) and IMG (**C**,**D**) cells were supplemented with different concentrations of zerumbone (1, 5, or 10 μM) for 30 min and then activated by LPS (50 ng/mL) for another 6 h. CXCL-10 (**A**,**C**) and CCL-2 (**B**,**D**) mRNA expression was analyzed using real-time PCR and normalized to β-actin. Data are presented as the mean ± SEM (*n* = 3). * *p* < 0.05, ** *p* < 0.01, *** *p* < 0.005 compared with the control group. # *p* < 0.05, ## *p* < 0.01 compared with the LPS alone.

We investigated the effect of zerumbone on the LPS-stimulated expression of proinflammatory mediators associated with M1-like macrophage/microglia polarization. The mRNA expression of CXCL-10 and CCL-2 was elevated in the mouse macrophages (Figure 4A,B) and IMG cells (Figure 4C,D) following LPS stimulation. Furthermore, zerumbone concentration-dependently decreased the LPS-induced increased expression of CXCL-10 (Figure 4A,C) and CCL-2 (Figure 4B,D) in both cell models.

**Figure 5:**
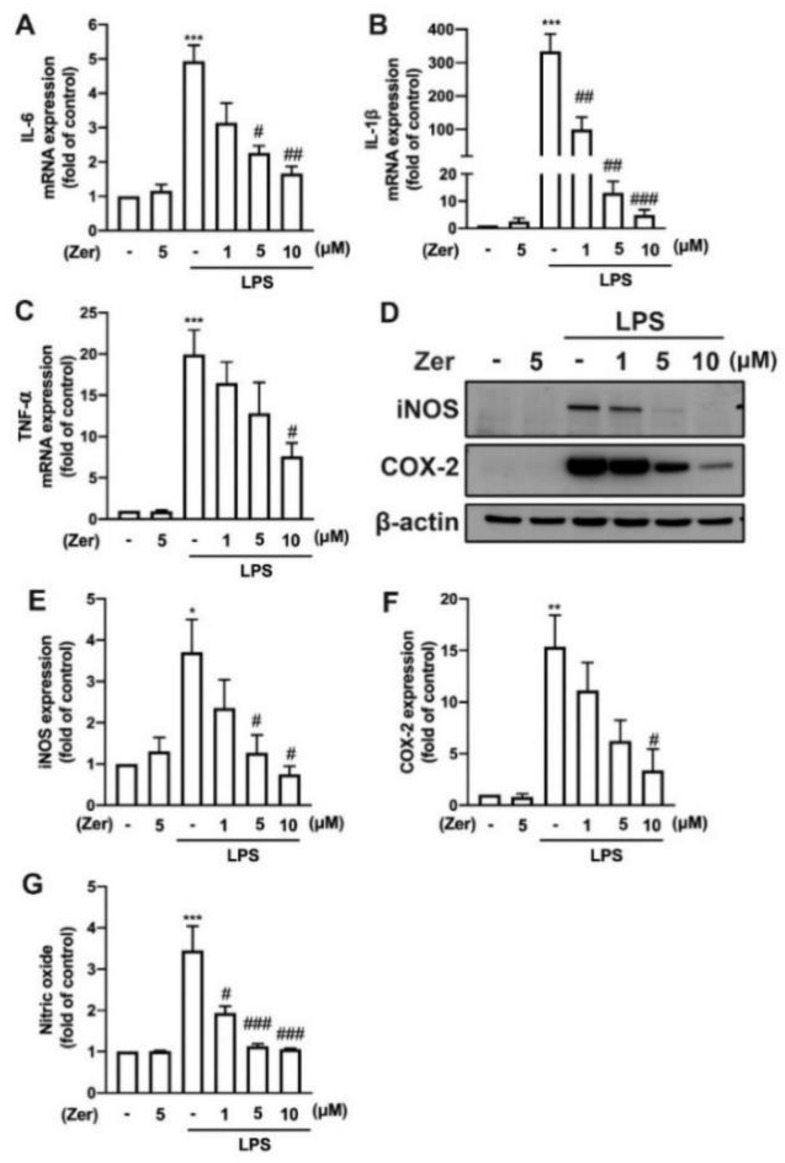
The expression of proinflammatory mediators in response to zerumbone in macrophages. RAW264.7 cells were supplemented with different concentrations of zerumbone (1, 5, or 10 μM) for 30 min and then stimulated with LPS (50 ng/mL) for another 6 h (**A**–**C**) or 24 h (**D**–**G**). Expressions of IL-6 (**A**), IL-1β (**B**), and TNF-α (**C**) mRNA were analyzed using real-time PCR and normalized to β-actin. (**D**) iNOS and COX-2 protein expressions were analyzed using Western blotting. Quantitative results are shown in (**E**,**F**). (**G**) The cultural supernatant was harvested for measuring NO production by NO assay. Each bar represents the mean ± SEM (*n* = 3 or 4). * *p* < 0.05, ** *p* < 0.01, *** *p* < 0.005 compared with the control group. # *p* < 0.05, ## *p* < 0.01, ### *p* < 0.005 compared with the LPS alone group.

**Figure 6:**
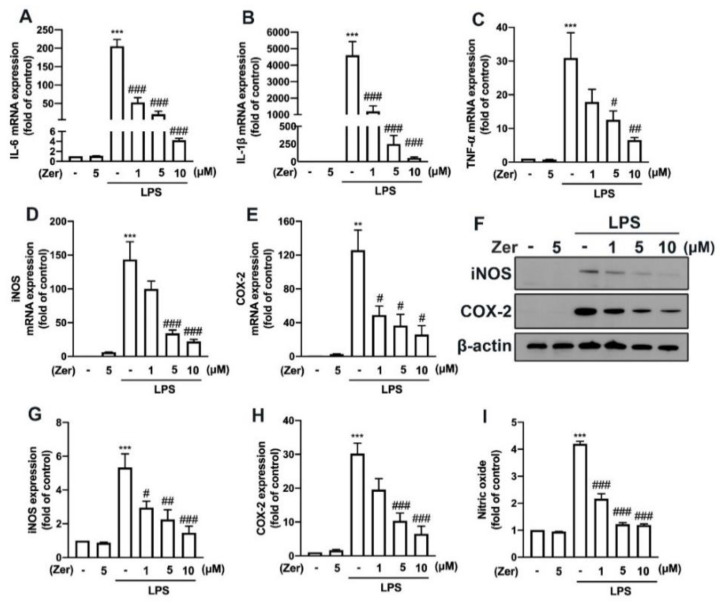
The expression of proinflammatory mediators in response to zerumbone in microglia. IMG cells were supplemented with different concentrations of zerumbone (1, 5, or 10 μM) for 30 min and administered with LPS (50 ng/mL) for another 6 h (**A**–**C**) or 24 h (**D**–**G**). IL-6 (**A**), IL-1β (**B**), TNF-α (**C**), iNOS (**D**), and COX-2 (**E**) mRNA expressions were analyzed using real-time PCR and normalized to β-actin. (**F**) iNOS and COX-2 protein expressions were analyzed using Western blotting. Quantitative results are shown in (**G**,**H**). (**I**) The cultural supernatant was harvested for measuring NO production by NO assay. Each bar represents the mean ± SEM (*n* = 3 or 4). ** *p* < 0.01, *** *p* < 0.005 compared with the control group. # *p* < 0.05, ## *p* < 0.01, ### *p* < 0.005 compared with the LPS alone group.

Additionally, zerumbone considerably and concentration-dependently reduced the LPS-induced upregulation of M1 polarization markers such as IL-6 (Figure 5A), IL-1β (Figure 5B), and TNF-α (Figure 5C) in macrophages. We further observed similar inhibitory effects of zerumbone on LPS-induced IL-6 (Figure 6A), IL-1β (Figure 6B), and TNF-α (Figure 6C) in microglial cells. Moreover, supplement with zerumbone attenuated LPS-stimulated expression of iNOS (Figure 5D,E) and COX-2 (Figure 5D,F) proteins in macrophages in a concentration-dependent manner. Zerumbone further inhibited LPS-induced NO production in macrophages (Figure 5G). Furthermore, zerumbone effectively reduced the mRNA expression of iNOS (Figure 6D) and COX-2 (Figure 6E) induced by LPS. Supplement with zerumbone attenuated the expression of iNOS (Figure 6F,G) and COX-2 (Figure 6F,H) proteins induced by LPS, as well as NO production, dose-dependently (Figure 6I). We did not observe any change in the expression of proinflammatory mediators in macrophages (Figure 4A,B and Figure 5) or microglia (Figure 4C,D and Figure 6) supplemented with zerumbone alone. The results suggest that supplement with zerumbone reversed LPS-activated macrophage and microglia polarization toward the M1 phenotype.

### 3.5. Zerumbone Promotes Endogenous Antioxidant Production and IL-10 Expression in Microglial Cells

**Figure 7:**
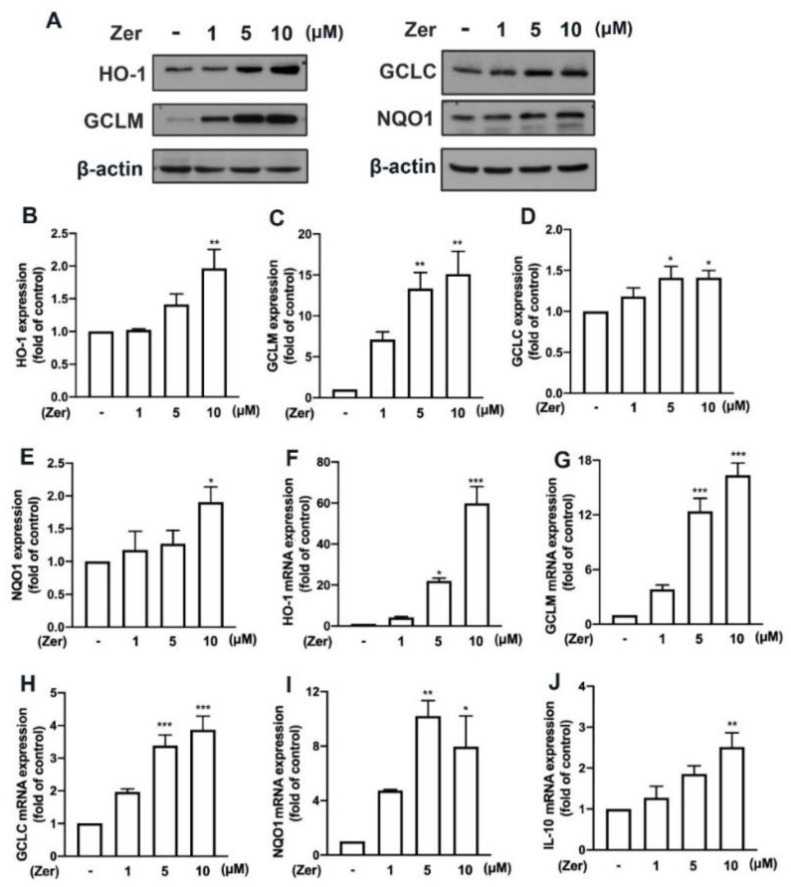
The expression of endogenous antioxidants and anti-inflammatory cytokines in response to zerumbone in microglia. (**A**) Different concentrations of zerumbone (1, 5, or 10 μM) were supplemented on IMG cells for 24 h. Protein expressions of HO-1, GCLM, GCLC, and NQO1 were evaluated using Western blotting. The quantitative results of HO-1 (**B**), GCLM (**C**), GCLC (**D**), and NQO1 (**E**) were determined by using ImageJ. Different concentrations of zerumbone (1, 5, or 10 μM) were supplemented on IMG cells for 6 h. HO-1 (**F**), GCLM (**G**), GCLC (**H**), NQO1 (**I**), and IL-10 (**J**) mRNA expressions were quantified using real-time PCR. Each bar represents the mean ± SEM (*n* = 3 or 4). * *p* < 0.05, ** *p* < 0.01, *** *p* < 0.001 compared with the control group.

Several naturally occurring compounds stimulate the production of endogenous antioxidants—such as heme oxygenase (HO)-1, glutamate–cysteine ligase modifier subunit (GCLM), glutamate–cysteine ligase catalytic subunit (GCLC), and NAD(P)H quinone oxidoreductase-1 (NQO1)—and promotes microglial polarization toward M2-like phenotypes that are beneficial for maintaining cellular redox homeostasis and are anti-inflammatory [18,36,37]. This study showed that supplement with zerumbone remarkably promoted the expression of the endogenous antioxidant proteins HO-1 (Figure 7A,B), GCLM (Figure 7A,C), GCLC (Figure 7A,D), and NQO1 (Figure 7A,E) in microglial cells. Moreover, the mRNA expression of HO-1 (Figure 7F), GCLM (Figure 7G), GCLC (Figure 7H), and NQO1 (Figure 7I) was upregulated following zerumbone supplement in microglial cells. Moreover, as shown in Figure 7J, zerumbone increased the expression of the M2 phenotype marker IL-10 in a dose-dependent manner. These data suggest that the antineuroinflammatory properties of zerumbone were modulated by the production of endogenous antioxidants.

### 3.6. AMPK and Akt/GSK3 Signaling Pathways Mediate Zerumbone-Stimulated Production of Endogenous Antioxidants in Microglial Cells

**Figure 8:**
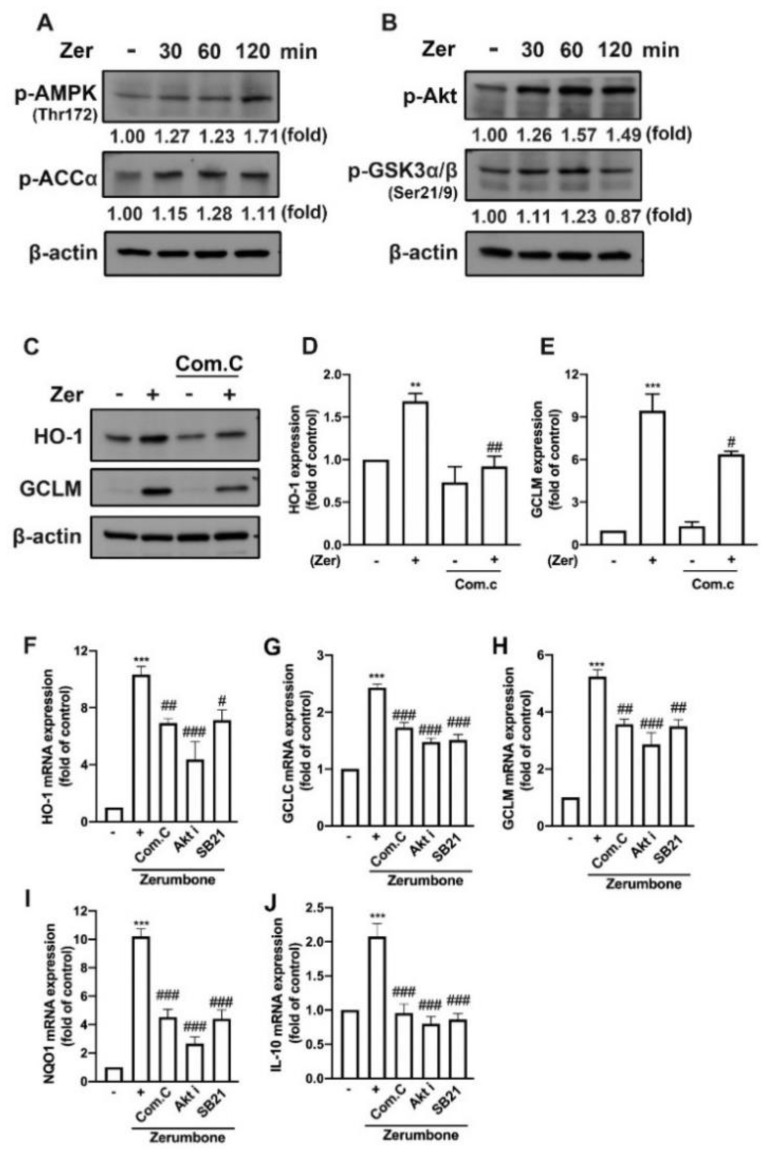
Zerumbone-induced endogenous antioxidant expression is mediated through AMPK and Akt/GSK3 signaling pathways. IMG cells were supplemented with zerumbone (10 μM) for 30, 60, or 120 min. The phosphorylation of AMPK and ACCα (**A**) and of Akt and GSK3α/β (**B**) were examined by Western blotting. AMPK inhibitor compound C (15 μM) was administered 30 min before supplemented with zerumbone (10 μM) for another 24 h. (**C**) HO-1 and GCLM protein expressions were detected by Western blotting, with quantitative data shown in (**D**) and (**E**). Compound C, Akt inhibitor (10 μM), or SB 216763 (SB21; 20 μM) were administered 30 min before supplemented with zerumbone for another 6 h. HO-1 (**F**), GCLC (**G**), GCLM (**H**), NQO1 (**I**), and IL-10 (**J**) mRNA expressions were determined using real-time PCR and normalized to β-actin. The quantitative results in bar graphs represent the mean ± SEM (*n* = 3 or 4). ** *p* < 0.01, *** *p* < 0.005 compared with the control group. # *p* < 0.05, ## *p* < 0.01, ### *p* < 0.005 compared with zerumbone alone.

Studies have shown that zerumbone activates AMPK signaling pathways and the downstream target of AMPK, acetyl-CoA carboxylase (ACC), contributing to a protective role in high-glucose-stimulated renal tubular cells [38] and high-fat-diet-induced obesity in mice [39]. Furthermore, zerumbone attenuated inflammatory responses in mice with acute lung injury [40] and macrophages [26] by modulating the Akt pathway. In the present study, the zerumbone supplement enhanced activation of the AMPK (Figure 8A) and Akt (Figure 8B) signaling pathways, as well as their downstream targets ACC (Figure 8A) and GSK3 (Figure 8B). In addition, supplement with an AMPK inhibitor (compound C) suppressed the expression of HO-1 (Figure 8C,D), GCLM (Figure 8C,E), and GCLC (Figure 8C) proteins in microglial cells supplemented with zerumbone. We further confirmed the involvement of the AMPK/Akt signaling pathways in the protective effects of zerumbone. As illustrated in Figure 8, the zerumbone-induced expression of endogenous antioxidant genes such as HO-1, GCLC, GCLM, and NQO1 (Figure 8F–I), as well as M2 phenotype marker IL-10 (Figure 8J), was inhibited by compound C, Akt inhibitor, and GSK3 inhibitor (SB21). These findings indicate that zerumbone promoted endogenous antioxidants and polarization toward M2 phenotypes by mediating the AMPK and Akt/GSK3 pathways in microglial cells.

## 4. Discussion

Zerumbone has been reported to modulate oxidative stress in several cancers, including breast cancer [18], nonsmall-cell lung cancer [36], and colon cancer [37]. Furthermore, it has been reported that zerumbone enhances the radiosensitivity and chemosensitivity of these malignancies. Notably, zerumbone has been reported to induce oxidative stress and cell apoptosis in protozoan parasites [38]. A recent report indicated that zerumbone protects against zearalenone-induced hepatotoxicity in mice by activating endogenous antioxidants, including glutathione and superoxide dismutase [39]. Moreover, zerumbone treatment also protected against oxidative stress in a mouse model of acute liver injury [40]. A few studies have reported that zerumbone exerts biological effects on the CNS. Treatment with zerumbone reversed scopolamine-induced memory impairments in rats [33] and social memory in triple transgenic Alzheimer’s disease (AD) mouse models [41]. In addition, the cotreatment of zerumbone with polyunsaturated fatty acids attenuated oxidative stress in the brain by increasing antioxidants and neurotrophins [42]. A recent study suggested that in APP/PS1 transgenic mice, zerumbone decreased the expression of proinflammatory cytokines, lessened the amount of β-amyloid accumulation, and reduced behavioral deficits, partly due to the production of IL-10 by activated microglia [34]. The present study confirmed the antioxidative and anti-inflammatory properties of zerumbone by demonstrating that zerumbone effectively reduced inflammation and oxidative stress in macrophages and microglia without any toxicity being incurred.

Production of lipocalin-2 may act as a signal during oxidative stress and inflammation. Lipocalin-2 is produced in the CNS in response to acute-phase brain injury, further triggering the inflammation-related chemokine CXCL10, which promotes the migration of astrocytes to injury sites [43,44]. Neutralization of lipocalin-2-attenuated neurological deficits and cerebral infarction by diminishing the expression of M1 macrophage polarization in the brain in a stroke-reperfusion injury mouse model [45]. Additionally, lipocalin-2-deficient mice had less hyperalgesia, M1 macrophage polarization, and macrophage inflammatory protein 2 production in response to complete Freund’s adjuvant. [46]. The level of lipocalin-2 was found to be increased in patients with AD [47] and Parkinson’s disease (PD) [48]; the patient’s pathophysiology was also aggravated along the lipocalin-2 levels. Moreover, a recent study considered lipocalin-2 to be a promising therapeutic target in the management of dementia [49]. In our previous study, we demonstrated that management of lipocalin-2 reduced astrocyte activation and improved cognitive functions, social avoidance, and anxiety-like behaviors [50]. Notably, one study [23] and our recent study [51] have reported that lipocalin-2 may be a regulator of M1 and M2 macrophage polarization. The present study demonstrated the role of zerumbone in decreasing the expression of lipocalin-2 and thereby improving the inflammatory response and tissue homeostasis in activated microglia and macrophages.

Macrophages respond to external and endogenous stimuli by switching their phenotypes to resolve inflammation and maintain immune defense and homeostasis [1]. One study demonstrated that in an experimental PD mouse model, an increase in M1 macrophages in the peripheral immune system triggered the expression of proinflammatory mediators such as iNOS, IL-1β, and TNF-α in brain, leading to neuronal cell death [52]. Notably, by depleting peripheral M1 macrophages and promoting M2 macrophages, T cell infiltration to the brain was reduced, thereby reducing brain inflammation, neuronal cell death, and behavioral deficits [52]. In experimental autoimmune encephalomyelitis (EAE), activated M1 microglia upregulated CCL-2, which facilitated the recruitment of circulating monocytes to the injured sites, as well as TNF and iNOS, which contributed to inflammation [53]. Overproduction of ROS in macrophages may trigger necrosis, which leads to the production of proinflammatory mediators and aggravates inflammation [54]. One study revealed that mitochondrial ROS generated by activated macrophages stimulated the expression of IL-1β, TNF-α, and CCL-2, thereby increasing the risk of developing high-fat-induced insulin resistance and atherosclerosis [55]. Moreover, increased levels of ROS were shown to trigger inflammation and cause apoptotic death of microglia under oxygen–glucose deprivation conditions [56]. Notably, NADPH oxidase (NOX)-1-derived ROS increased the expression of lipocalin-2 in colon epithelial cells stimulated with TNF-α and IL-17 [57]. Accordingly, NOX-1-knockout mice exhibited reduced expression of lipocalin-2 along with improved colon condition [57]. Treatment with zerumbone was found to diminish ROS production and protect cells from high-glucose-stimulated pancreatic β cells [58]. The present study supported previous studies that zerumbone could protect microglia and macrophages by regulating the production of ROS and expression of proinflammatory mediators.

Macrophages require ROS during the uptake and clearance of dying cellular parts. ROS control the phagocytic activity of macrophages [59]. Increased intracellular production of ROS was reported to enhance the phagocytosis of macrophages [60]. Phagocytosis by microglia is associated with neurodegenerative diseases [61]. The roles of microglia and phagocytosis in different stages of neurodegenerative disorders remain unknown. Phagocytosis by activated microglia can be beneficial in terms of the clearance of Aβ in AD [62]. However, microglia may be detrimental to the pathophysiology of AD because they stimulate neurotoxins [61]. Similarly, a study suggested that there is a delicate balance between activated microglial damage of myelin-generating cells and activated microglial repair and support of neurogenesis in multiple sclerosis [63]. Thus, maintaining macrophage homeostasis and phagocytic activity is beneficial for disease management. Notably, lipocalin-2-deficient mice exhibited lower phagocytic activity than wild-type mice [64]. A recent study suggested that lipocalin-2 regulated myelin phagocytosis in an ischemic stroke mouse model [65]. The present study demonstrated the regulatory effects of zerumbone on proinflammatory-stimulus-triggered lipocalin-2 expression and microglial phagocytic activity. On the other hand, zerumbone is also reported to exert anti-inflammatory effects through pathways other than lipocalin-2, such as the Akt-NFkB pathway and NLRP3 inflammasome [40,66], indicating that zerumbone may augment inhibitory effects on M1/M2 polarization, cytokine production, and ROS formation.

The production of IL-10 is facilitated by the protein expression of M2 phenotypes, which suppress inflammation and restore homeostasis [67]. Treatment with recombinant IL-10 considerably decreased M1 macrophage polarization in LPS-activated microglia [68]. Moreover, IL-10-deficient mice exhibited a decreased inflammatory response and persistent ischemia, suggesting the role of IL-10 in attenuating local inflammatory responses [68]. IL-10 overexpression was found to be beneficial for the treatment of several neurodegenerative diseases—including SCI [16], EAE [69], and AD [70]—by reducing the expression of proinflammatory mediators and improving neurological functions. Evidence was found that IL-10 signaling is correlated with the expression of HO-1 [71]. The induction of endogenous antioxidants, such as glutathione-S-transferases, regulates inflammatory responses [72]. Our previous studies have demonstrated that treatment with naturally occurring compounds—quercetin [51], paeonol [73], fisetin [74], and caffeic acid phenethyl ester [75]—induces the expression of HO-1 and promotes the polarization of macrophages toward the M2 phenotype that inhibits proinflammatory responses in microglia. In addition, zerumbone has been reported to upregulate the expression of HO-1 and γ-glutamyl cysteine ligase in human keratinocyte cells [76]. Zerumbone enhances GSK3β phosphorylation in meningioma cells [75,77]. A recent study reported that zerumbone supplement activates the PI3/Akt signaling pathway and upregulates the expression of endogenous antioxidants against hepatotoxicity [39]. Additionally, stimulation of endogenous antioxidants is regulated by activation of the AMPK/Akt signaling pathway [78,79]. The present study demonstrated that zerumbone significantly stimulated the expression of endogenous antioxidants and M2 macrophage markers involving AMPK/Akt and Akt/GSK3β signaling pathways, resulting in zerumbone having antioxidant and protective roles in microglia and macrophages.

The limitation of this study includes that no result was obtained from in vivo experimental models. If additional animal models were carried out, we could provide substantial information considering the effectiveness of zerumbone and improve our understanding of zerumbone under systemic conditions. From our established in vivo model, we found that LPS-induced inflammation provokes IL-6 and TNF-α production in mouse brain microglia [80]. LPS also causes impaired motor balance and coordination function in mice [36]. Moreover, LPS injection also induces microglia to change their normal ramified morphology into an activated hypertrophic form [81]. In this study, we attempted to focus on the effects of zerumbone on M1/M2 polarization and the ability of phagocytosis, and we chose macrophages and microglia cell lines to clarify the effect of zerumbone.

## 5. Conclusions

This study demonstrated the potential role of zerumbone in reducing the expression of lipocalin-2- and M1-associated inflammatory responses, including the overexpression of proinflammatory cytokines (IL-1β, IL-6, and TNF-α), chemokines (CCL-2 and CXCL-10), iNOS, NO, and COX-2 in activated microglia and macrophages. Supplement with zerumbone was discovered to effectively reduce ROS production stimulated by H_2_O_2_, ROO•, and HO•. The phagocytic activity of microglial cells triggered by proinflammatory stimuli was also lower in cells subjected to zerumbone supplement. The expression of IL-10 in both microglia and macrophages was increased following supplementation with zerumbone. Notably, we discovered that zerumbone increased the expression of HO-1, GCLM, GCLC, and NQO1 by regulating the AMPK and Akt/GSK3β signaling pathways. This study suggests that zerumbone could be a potential supplement for inflammatory diseases in both the CNS and peripheral systems due to its ability to regulate cellular redox homeostasis and macrophage polarization.

## Data Availability

Data are available from the corresponding author on reasonable request.

## Supplementary Materials

The following supporting information can be downloaded at: https://www.mdpi.com/article/10.3390/nu14245402/s1, Figure S1: effects of zerumbone on viability of macrophages, Figure S2: effects of zerumbone on viability of microglia.
