# Supplementary material for: Role of Zerumbone, a Phytochemical Sesquiterpenoid from Zingiber zerumbet Smith, in Maintaining Macrophage Polarization and Redox Homeostasis
Source: Nutrients. 2022 Dec 19;14(24):5402. doi: 10.3390/nu14245402 (PMC9783216; doi:10.3390/nu14245402)
Supplement: Supplementary file 1 [file nutrients-14-05402-s001.zip › nutrients-2039874-supplementary.pdf]

Role of zerumbone in maintaining macrophage polarization and redox homeostasis

Wei-Lan Yeh, Bor-Ren Huang, Guan-Wei Chen, Vichuda Charoensaensuk, Liang-Yo Yang, Dah-Yuu Lu, and Chingju Lin

Supplementary Figure legends

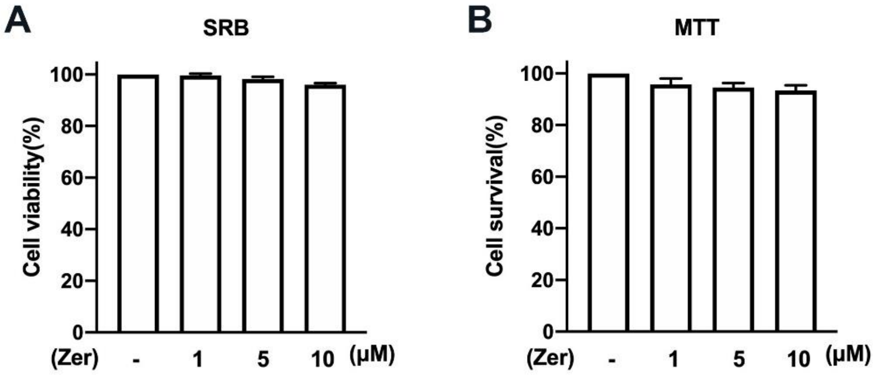

Supplementary Figure S1. Effects of zerumbone on viability of macrophages. RAW264.7 cells were incubated with various concentrations (1, 5, or 10 μM) of zerumbone for 24 h. Cell viability was then determined using the sulforhodamine B (SRB) (**A**) and 3-[4,5-dimethylthiazol-2-yl]-2,5 diphenyl tetrazolium bromide (MTT) (**B**) assays. The data represent the mean ± SEM (n = 4).

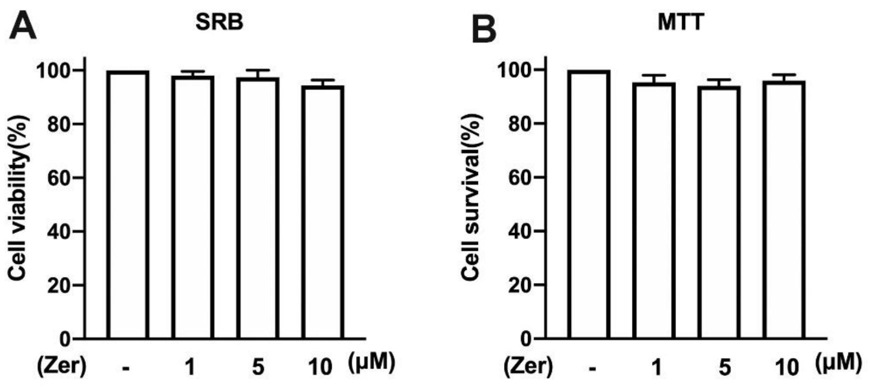

Supplementary Figure S2. Effects of zerumbone on viability of microglia. IMG cells were incubated with various concentrations (1, 5, or 10 μM) of zerumbone for 24 h. Cell viability was then determined using SRB (**A**) and MTT (**B**) assays. The data represent the mean ± SEM (n = 4).
